# Efficacy and Safety of LASIK Combined with Accelerated Corneal Collagen Cross-Linking for Myopia: Six-Month Study

**DOI:** 10.1155/2016/5083069

**Published:** 2016-09-04

**Authors:** Ying Wu, Lei Tian, Li-qiang Wang, Yi-fei Huang

**Affiliations:** ^1^Department of Ophthalmology, Chinese PLA General Hospital, Beijing 100853, China; ^2^Beijing Institute of Ophthalmology, Beijing Tongren Eye Center, Beijing Tongren Hospital, Capital Medical University, Beijing Ophthalmology & Visual Sciences Key Laboratory, Beijing 100730, China

## Abstract

This was a prospective controlled clinical trial. 48 myopia patients (96 eyes) were included in this study. After LASIK, accelerated corneal collagen cross-linking (ACXL) was used for myopia treatment. During 6-month follow-up, the results of LASIK-ACXL treatment were studied and compared to the LASIK-only procedure. The results showed that no statistically significant differences in UDVA, CDVA, MRSE, *K* mean, pachymetry, or ECD were found between the two groups at the visit after 6 months of follow-up (all *P* > 0.05). At 6 months postoperatively, 2 eyes lost one or more lines of visual acuity in the LASIK-ACXL group, whereas all LASIK-only treated eyes had a stable CDVA.* In vivo* confocal microscopy showed a decrease of keratocyte density and appearance of honeycomb-like structures in the anterior residual stroma bed; the changes were similar but more pronounced following LASIK-only. None of the cases in both groups developed signs of significant keratitis, regression, or ectasia during the 6-month follow-up. LASIK-ACXL can effectively correct refractive error in patients with myopia, with no significant complications during 6-month follow-up, indicating stability and morphologic change similar to those with LASIK-only treatment.

## 1. Introduction

In the past 20 years, corneal refractive surgeries have developed rapidly; laser-assisted* in situ* keratomileusis (LASIK) has become the most commonly used corneal refractive surgical procedure to treat myopia and astigmatism worldwide [1]. The principle of LASIK is to ablate corneal stroma by laser technique, altering central corneal curvature to correct refractive error and focus incident light on the retina [2]. While the safety and efficacy of LASIK have been established, rare but serious complications can occur, such as iatrogenic keratectasia [3]. Postoperative ectasia is a result of corneal alteration by severing of stromal lamellae through the creation of the LASIK flap, combined with stromal ablation, which together can weaken corneal biomechanical properties and affect the corneal stability [4]. The clinical presentation of ectasia includes increasing myopia and/or astigmatism, progressive topographic changes, progressive corneal thinning, and a loss of corrected distance visual acuity (CDVA).

The corneal collagen cross-linking (CXL) procedure increases covalent bonding between or within collagen fibers in the corneal stroma, thus increasing corneal stiffness and improving corneal biomechanical stability [5]. The long-term efficacy of CXL has already been demonstrated in the treatment of primary and secondary (post-LASIK and post-RK) corneal ectasia [6–8]. Since ectasia is one of the serious complications after LASIK, some researchers propose a clinical application of ACXL-assisted LASIK (LASIK-ACXL), which may be considered a prophylactic biomechanical treatment, stiffening the intermediate corneal stroma with the aim of reducing the risk of corneal ectasia and stabilizing the clinical results of refractive surgery. Recently, ACXL was performed concurrently with LASIK in a small group of patients in order to prevent corneal ectasia after LASIK treatment, reporting good clinical results [7]. The results of this pilot case series suggest that an evaluation of a larger study cohort is warranted to establish the utility of this treatment [9, 10].

The aim of this prospective study was to evaluate the efficacy and safety of LASIK-ACXL as compared to LASIK alone at 1, 3, and 6 months postoperatively. Visual acuity, refractive error, corneal morphology, endothelial cell density (ECD), and the changes of corneal microstructure were recorded and compared with those in the LASIK-only treated eyes.

## 2. Materials and Methods

### 2.1. Subject Recruitment

96 eyes of 48 consecutive patients who were scheduled for LASIK surgery from March 2015 to January 2016 in the Department of Ophthalmology Refractive Center, Chinese PLA General Hospital (Beijing, China), were enrolled in this prospective comparative study. Half of the patients underwent only LASIK treatment (LASIK-only group) and the other half were treated with CXL and LASIK (LASIK-ACXL group). They were randomized into the two groups. All participants signed an informed consent form in accordance with the tenets of the Declaration of Helsinki. This study also received institutional review board approval of Chinese PLA General Hospital, Beijing, China.

Inclusion criteria included age ≥18 years, preoperative spherical refractive error from −1.00 D to −11.00 D, refractive cylinder <−5.00 D, stable refractive error for more than 2 years, and willingness and ability to comply with postoperative care. Exclusion criteria included central corneal thickness (CCT) less than 480 *μ*m, predicted postoperative residual stroma bed thickness of less than 280 *μ*m, and patients with any other eye disease, external injury history, systemic diseases, or any other history of ocular surgery. Participants were instructed to remove soft contact lenses at least 2 weeks or rigid contact lenses at least 4 weeks before the screening examination.

### 2.2. Preoperative Examination

All subjects underwent a systematic ocular examination preoperatively and follow-up visits at 1, 3, and 6 months. The following were assessed at each visit: UDVA, corrected distance visual acuity (CDVA), manifest refraction, cycloplegic refraction, intraocular pressure (IOP), slit-lamp microscopy, fundus examination, corneal topography (Allegro Topolyzer, Wavelight AG, Germany), corneal tomography (Pentacam, Oculus Optikgeräte GmbH, Wetzlar, Germany), and ECD (SP-3000, Topcon, Japan). The pachymetry and topographic mean *K* (*K* mean) values were measured using Pentacam.

Confocal laser scanning microscopy with a retina tomograph (HRT III with Rostock Cornea Module, Heidelberg Engineering, Heidelberg, Germany) was used to observe the morphologic changes in the corneal stroma. The physician who evaluated the corneal tissue morphology on the images was blinded to which eye had ACXL.

### 2.3. LASIK Procedure

Conventional bilateral LASIK was performed using a Wavelight FS200 (Alcon Laboratories, Inc.) for flap creation with thickness of 90 *μ*m or 110 *μ*m and a Wavelight EX500 excimer laser (Alcon Laboratories, Inc.) for refractive correction. All laser parameters and laser nomograms for LASIK were applied according to the clinic's typical LASIK protocol.

### 2.4. LASIK Combined with ACXL Procedure

Patients were prepared using the same process of flap creation as for LASIK-only group. After the ablation of the corneal stroma, the single-use VibeX Xtra riboflavin drops (0.25% riboflavin; Avedro, Waltham, Massachusetts, USA) were instilled onto the exposed stromal bed for 90 seconds. Then balanced salt solution was used to flush remaining riboflavin from the stromal bed, the flap was then repositioned, and the KXL system (Avedro, Waltham, Massachusetts, USA) was used to apply 90 seconds of continuous light illumination at 30 mW/cm^2^ (total exposure dose of 2.7 J/cm^2^) over the closed flap, according to manufacturer's recommendations.

### 2.5. Postoperative Protocol

Postoperative follow-up was at 1 day and 1 week after the surgery. Complete ophthalmic evaluations were conducted at 1, 3, and 6 months postoperatively. Postoperative regimens were prescribed for both eyes as follows: 0.1% fluorometholone eye drop, four times per day, and sodium hyaluronate eye drop, four times per day.

### 2.6. Statistical Analysis

Statistical analysis was performed using SPSS 17.0 (SPSS Inc., Chicago, Illinois, USA). The Kolmogorov-Smirnov test was used to check for normal distribution of quantitative data, provided here as the mean ± standard deviation (SD). The significance of the change from preoperation to postoperation was evaluated using the paired *t*-test. Independent two-sample *t*-tests were used to compare data between the two groups. If the data were not normally distributed, the Wilcoxon rank-sum test was performed. *P* values less than 0.05 were considered as statistically significant.

## 3. Results

### 3.1. Demographics

At 1, 3, and 6 months, 96 eyes were observed. The LASIK-ACXL group included 25 males and 23 females with a mean age of 24.63 ± 3.85 years (range: 18–33 years). The LASIK-only group included 20 males and 28 females aged 18–34 years, with mean age of 25.33 ± 4.06 years. There was no significant difference in age (*P* = 0.383) or gender (*P* = 0.306) distribution between the two groups. The parameters and data before operation and at 1, 3, and 6 months after operation are shown in Table 1.

### 3.2. Visual Acuities

**Figure 1:**
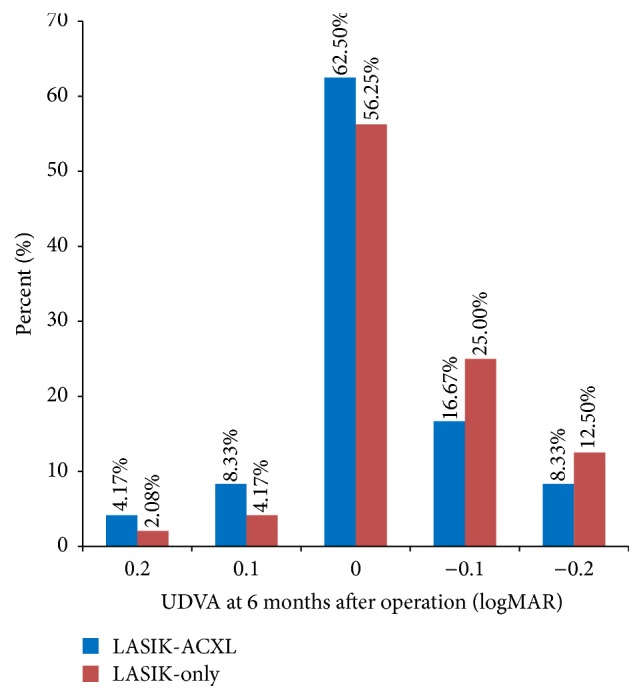
Distribution of uncorrected distance visual acuity (UDVA) at cumulative 6 months after operation. Forty-two (87.5%) of 48 eyes in the LASIK-ACXL group and 45 (93.75%) of 48 eyes in the LASIK-only group had 0 logMAR or better at 6 months.

There were no statistically significant differences in UDVA and CDVA between the two groups from preoperation to postoperation except in the UDVA (*P* < 0.001) and CDVA (*P* = 0.005) at 1 month after operation. All eyes had emmetropia as the target refraction. At 6 months after operation, the efficacy index (mean postoperative UDVA/mean preoperative CDVA) was 1.02 ± 0.18 in LASIK-ACXL group and 1.06 ± 0.25 in the LASIK-only group.  Figure 1 shows the UDVA of LASIK-ACXL group and LASIK-only group at 6 months after operation.

**Figure 2:**
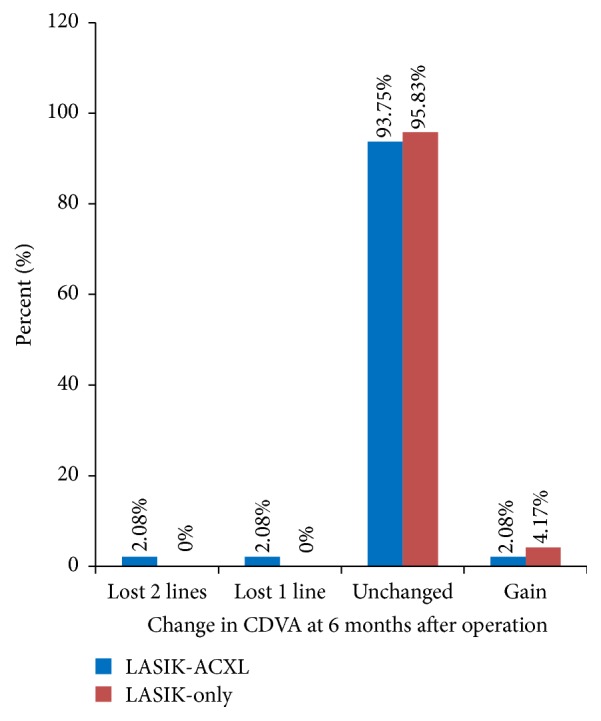
The percentage of eyes in which there was a gain or loss of logMAR visual acuity lines compared with preoperative levels for different postoperative periods. At 6 months, 2 eyes (4.17%) in the LASIK-ACXL group lost one or more lines, whereas all LASIK-only treated eyes had unchanged corrected distance visual acuity (CDVA).

The safety index (mean postoperative CDVA/mean preoperative CDVA) was 1.09 ± 0.32 at 6 months in the LASIK-ACXL group and 1.15 ± 0.23 in the LASIK-only group.  Figure 2 shows the percentage of eyes in which there was a gain or loss of logMAR visual acuity lines compared with preoperative levels at 6 months.

### 3.3. Refractive Diopter Results

**Figure 3:**
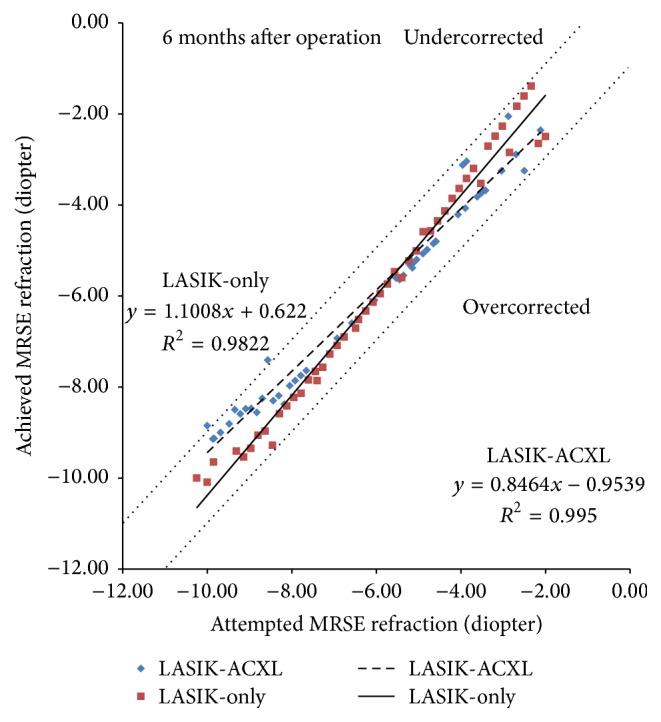
Attempted manifest refraction spherical equivalent (MRSE) refractive change plotted against the achieved MRSE refractive change. At 6 months, 46 (95.83%) eyes in the LASIK-ACXL group and 48 (100%) eyes in the LASIK-only group had MRSE within ±1.0 diopter.

Figure 3 shows a scatter plot and linear regression analysis of the attempted MRSE refractive change plotted against the achieved MRSE refractive change postoperatively. There was no significant difference between the regression coefficients of the LASIK-ACXL and LASIK-only groups at 6 months (*P* = 0.074).

### 3.4. Keratometry

**Figure 4:**
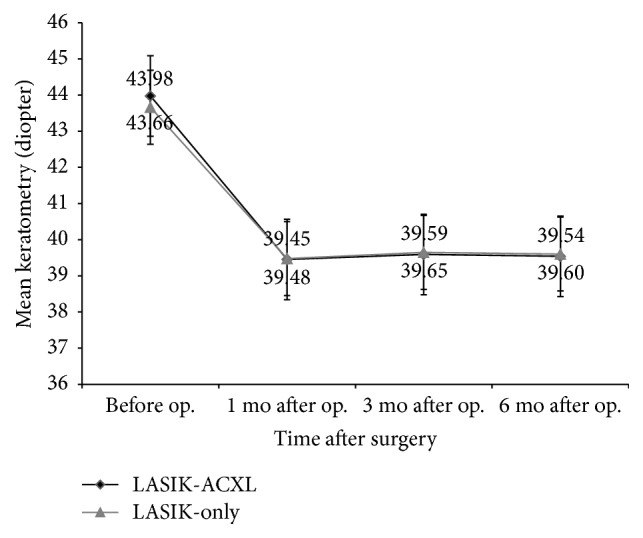
*K* mean changes before operation and at 1, 3, and 6 months after operation.

There was no statistically significant difference in the topographic *K* mean value between the two groups through the 6-month follow-up. The change of *K* mean values from 1 month to 6 months after operation in the LASIK-ACXL group was 0.09 ± 1.24 D and in LASIK-only it was 0.13 ± 1.45 D, respectively (*P* = 0.887).  Figure 4 shows *K* mean changes in each postoperative follow-up period, demonstrating the stability of the results.

### 3.5. Endothelial Cell Density

ECD in the two groups was not statistically different at 6 months after operation compared to the preoperative data (LASIK-ACXL, *P* = 0.295; LASIK-only, *P* = 0.32).

### 3.6. Microstructure Changes by* In Vivo* Confocal Microscopy

**Figure 5:**
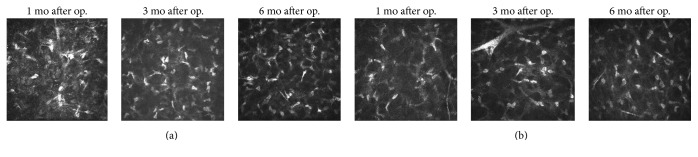
*In vivo* confocal images of the changes at the anterior residual stromal bed after LASIK-ACXL (a) and LASIK-only (b).

In the confocal scans, the interface between flap and residual stroma bed was hypocellular and well distinguished, containing numerous hyperreflective microspots or bright particles. At one month after operation, the keratocyte density decreased and honeycomb-like structures appeared in the anterior residual stroma bed, until a depth of about 60 *μ*m over the interface, in both LASIK-ACXL and LASIK-only groups. These changes were similar but more pronounced following LASIK-ACXL. At 6 months, the anterior residual stroma bed structure was almost restored to the preoperative status in both groups. For both treatment groups, the posterior residual stroma bed appeared to have not been affected, and no significant decline in endothelial cells was detected during the follow-up period.  Figure 5 shows confocal images of the changes after LASIK-ACXL and LASIK-only at the anterior residual stromal bed.

### 3.7. Postoperative Complications

Postoperatively patients experienced different irritations such as photophobia, lacrimation, and foreign-body sensation. The majority of these symptoms reduced or disappeared 1-2 days postoperatively. During the 6-month follow-up, all patients gained good corneal flap alignment with no complication such as haze, infectious, or noninfectious keratitis or other related complications.

## 4. Discussion

Iatrogenic keratectasia is one of the serious complications after LASIK surgery, also known as secondary keratoconus. The clinical symptoms including progressive, noninflammatory corneal thinning and ectasia lead to regression of refractive effect, increasing astigmatism and decreasing CDVA [4]. It is reported that the changes of biological and mechanical properties of the cornea can induce optical and morphological changes on the cornea. Despite the low morbidity rate of LASIK ectasia, the consequence is serious. Once it occurs, it causes disastrous clinical outcomes [11]. Despite advancements in refractive surgery, risk factors for development of post-LASIK ectasia remain unclear and may be missed in screening evaluation [12]. In addition, young patients with high myopia may have a greater risk of developing delayed ectasia after LASIK surgery [13]. Based on these studies, there is an urgent need for clinical methods to stabilize postoperative refractive status and corneal mechanical properties of patients with LASIK surgery.

It has been demonstrated that CXL can enhance corneal biomechanical properties. According to a long-term follow-up study by Frederik et al. [14], the majority of eyes with keratoconus treated by CXL maintained stability 10 years after the surgery. The traditional CXL process uses lower ultraviolet irradiance (3 mW/cm^2^), which increases the length of treatment time (30 min). Both doctors and patients hope to reduce therapy time and increase comfort and improve work efficiency of doctors. Rocha et al. [15] put forward the concept of “accelerated cross-linking” in 2008. Then Kanellopoulos [16, 17] started the research on the method of CXL combined with PRK in treating keratoconus; it is also known as “Athens Protocol.” In 2012, Kanellopoulos published 43 consecutive sequential LASIK-ACXL procedures, with higher fluence (irradiance: 10 mW/cm^2^; 370 nm wavelength ultraviolet-A light) applied for 3 minutes. The average follow-up duration was 3.5 years (1–4.5 years). The result showed patients had good logMAR vision without complications such as a decrease in ECD, occurrence of corneal ectasia, or refractive regression. The authors concluded that prophylactic ACXL for high-risk LASIK cases appeared to be a safe effective adjunctive treatment for refractive regression and potential ectasia [10]. A more efficient ACXL method is taken (irradiance: 30 mW/cm^2^; irradiation time: 90 s) in this study. At 6 months of follow-up, we found that LASIK-ACXL is effective and safe in the treatment of myopia, with similar recovery of the LASIK-only treatment method. Morphological changes observed by confocal microscopy, such as a decrease in keratocyte density and appearance of honeycomb-like structures in the anterior residual stromal bed, are similar to those observed after ACXL surgery [18, 19]. The microscopy changes were similar but more pronounced following LASIK-only. At 6 months, the anterior residual stroma bed structure was almost fully restored to the preoperative status in both groups.

Postoperative visual recovery is an important index to evaluate the operation efficacy. The efficacy and safety indices were comparable with the studies for the LASIK-only procedure by Chan et al. [20] and LASIK-ACXL by Tomita et al. [21]. When comparing proportions of eyes with UDVA of 0 logMAR or better, we found results similar to those of previous studies with LASIK-only [22, 23] and LASIK-ACXL [21]. One week after the procedure, UCVA and CDVA in the LASIK-ACXL group fluctuated; this may have been related to corneal edema and inflammatory reaction caused by ACXL. In the LASIK-ACXL treatment group, appearance of honeycomb-like structures and keratocyte activation were observed by confocal microscopy in the anterior residual stroma bed, which indicated mild corneal edema and inflammatory reaction. All patients reported that they were satisfied with both treatments. Concerning emmetropia as the target in this study, both LASIK-ACXL and LASIK-only groups had favorable tendency toward emmetropia at 6 months. In a study with LASIK-ACXL and LASIK-only, Tomita et al. [21] reported 12-month results in which eyes in both groups were close to emmetropia, similar to our results.

In this current study, there were no significant differences in refractive error between the LASIK-ACXL and LASIK-only groups postoperatively. Our results are promising and comparable with those published by Tomita et al. [21] who focused on LASIK-ACXL and LASIK-only methods and by Gertnere et al. [24] and Chan et al. [20] who both studied LASIK-only.

*K* mean decreased significantly in both LASIK-ACXL and LASIK-only groups and remained stable at follow-up visits at 1, 3, and 6 months, with no statistical differences from 1 to 6 months after operation between the two groups. The steady state of keratometers after surgeries indicated that both surgical methods can effectively improve the visual function of patients and maintain stability of corneal morphology in the early postoperative period, but a longer follow-up period is essential to further verify the stability of these results.

A potential risk of CXL is that it may induce endothelial cell damage. The ultraviolet irradiation energy of the traditional CXL procedure is 5.4 J/cm^2^, which is far lower than the threshold to induce corneal endothelial cell loss, iris, crystalline lens, or retinal damage [25]. Although riboflavin saturation in the residual stromal bed in the LASIK-ACXL treatment is relatively thin, the ultraviolet irradiation energy is only 2.7 J/cm^2^, which is also lower than the threshold for corneal endothelial cell damage. Compared with the preoperative values, the ECD of LASIK-ACXL group showed no obvious change after 6 months, pointing out that the combined surgery has no significant effect on postoperative endothelial cells. Many studies [19, 26] have evaluated the endothelium after refractive surgery or CXL, finding little or no change with no clinically significant reduction in ECD. However, with all corneas having pachymetry of more than 400 mm during CXL treatment and no statistically significant difference in the change in ECD between the LASIK-only group and the LASIK-CXL group being found, the lack of change in ECD was expected in our study. No complications, except haze in two eyes, such as refractive regression, corneal expansion, and infectious or noninfectious keratitis, appeared during the 6-month postoperative follow-up, proving that LASIK-ACXL surgery for myopia is safe and effective in the short-term follow-up.

*In vivo* confocal microscopy was used to observe the microstructural changes over time after LASIK. Keratocyte apoptosis and honeycomb-like stromal edema in the anterior residual stroma bed in the early postoperative period were seen after LASIK-ACXL and LASIK-only. It was more pronounced in the LASIK-ACXL group, although the microscopy changes were similar. Tomita et al. [21] also found similar results. Some studies have demonstrated the regeneration of corneal keratocytes starting from the second or the third month after CXL [18, 27]. In our study, the keratocyte repopulation, showing as a hyperreflective structure, was observed in both groups 3 months after surgery. At 6 months after operation, the anterior residual structures have basically recovered to preoperative status.

However, the current study had a few limitations. This was a short-term follow-up study. It is not possible to determine the longitudinal change of LASIK-ACXL treatment for myopia correction. The sample size of this study was relatively small and therefore the results should be interpreted cautiously. Further studies with a larger sample size and a longer term follow-up period are required to corroborate our results.

In summary, there were no statistically significant differences in visual acuity, refraction, keratometry, and ECD between the LASIK-ACXL group and the LASIK-only group. Confocal microscopy showed that the keratocyte density decreased and honeycomb-like structures appeared in the anterior residual stroma bed in both groups at one month after operation and they were less pronounced over time during follow-up. Along with the rapid development of ACXL technology, the safety of LASIK surgery, efficacy, and patient selection will further improve.

## Figures and Tables

**Table 1:** The differences in parameters before operation and 1, 3, and 6 months after operation.

Parameters	Before operation	After operation			*P* value	*P*′ value
		1 month	3 months	6 months		
*UCVA (logMAR)*						0.734
LASIK-ACXL	1.06 ± 0.20	−0.06 ± 0.08	−0.14 ± 0.06	−0.17 ± 0.04	<0.001	
LASIK-only	1.00 ± 0.21	−0.12 ± 0.05	−0.15 ± 0.07	−0.18 ± 0.04	<0.001	
*CDVA (logMAR)*						0.176
LASIK-ACXL	−0.17 ± 0.06	−0.14 ± 0.06	−0.17 ± 0.07	−0.18 ± 0.03	0.134	
LASIK-only	−0.18 ± 0.06	−0.17 ± 0.04	−0.17 ± 0.04	−0.18 ± 0.03	0.007	
*Sphere (D)*						0.063
LASIK-ACXL	−6.17 ± 1.89	0.15 ± 0.45	0.20 ± 0.45	0.18 ± 0.18	<0.001	
LASIK-only	−5.61 ± 2.81	0.10 ± 0.27	0.13 ± 0.52	0.14 ± 0.16	<0.001	
*Cylinder (D)*						0.582
LASIK-ACXL	−0.80 ± 1.00	−0.03 ± 0.22	−0.05 ± 0.19	−0.09 ± 0.24	<0.001	
LASIK-only	−0.65 ± 0.99	−0.02 ± 0.34	−0.03 ± 0.27	−0.04 ± 0.18	<0.001	
*MRSE (D)*						0.313
LASIK-ACXL	−6.54 ± 2.03	0.10 ± 0.58	−0.10 ± 0.43	−0.05 ± 0.33	<0.001	
LASIK-only	−5.95 ± 2.33	−0.05 ± 0.69	−0.05 ± 0.44	−0.02 ± 0.42	<0.001	
*Mean K (D)*						0.927
LASIK-ACXL	43.98 ± 1.69	39.45 ± 1.54	39.59 ± 1.68	39.54 ± 1.67	<0.001	
LASIK-only	43.66 ± 1.78	39.48 ± 1.84	39.65 ± 2.01	39.60 ± 2.11	<0.001	
*TCT (µm)*						0.844
LASIK-ACXL	524.52 ± 25.95	449.76 ± 41.56	440.19 ± 29.35	438.42 ± 27.40	<0.001	
LASIK-only	525.56 ± 25.99	440.64 ± 33.65	435.14 ± 26.99	437.38 ± 24.37	<0.001	
*ECD (cell/mm* ^*2*^)						0.869
LASIK-ACXL	2732.38 ± 110.37	2809.64 ± 145.88	2712.49 ± 128.34	2698.17 ± 137.09	0.295	
LASIK-only	2726.75 ± 107.45	2763.45 ± 129.76	2796.57 ± 139.18	2702.96 ± 136.95	0.320	

*P* value: compared between preoperation and 6 months after operation.

*P*′ value: compared between two groups at 6 months after operation.

ACXL, accelerated corneal collagen cross-linking; UDVA, uncorrected distance visual acuity; CDVA, corrected distance visual acuity; MRSE, manifest refraction spherical equivalent; *K*, keratometry; TCT, thinnest corneal thickness; ECD, endothelial cell density.
